# Obsessive-Compulsive Disorder as a Risk Factor in Patients with Implantable Cardioverter Defibrillator

**Published:** 2011-07-01

**Authors:** K Banihashemian, M K Fakhri, M Moazzen

**Affiliations:** 1 Jahrom Payam-e-Nour University, Jahrom, Iran; 2 Islamic Azad University, Sari branch, Sari, Mazandaran, Iran; 3 Khavaran Payam-e-Nour University, Iran

**Keywords:** OCD, Risk factor, ICD, Coronary events, Iran

Dear Editor,

Psychological factors in patients after heart surgery have produced many researches in the past decade[1][2][3][4]. Most of the past studies have concerned with investigating the effect of depression and anxiety on the outcome of cardiac problems such as coronary artery bypass surgery [5][6], but the present study concerned with: 1) investigating distress in patients after implantable cardioverter defibrillator (ICD); and 2) investigating relationship between psychological factors and coronary events such as myocardial infarction, angina pectoris and cardiac death.

Thirty six patients (21 men and 15 women, between age of 50-78 years), that underwent recent (one month earlier) ICD in Tehran Heart Hospital (Iran) were enrolled. Patients were evaluated both at the initial evaluation and one year after surgery at follow-up visit (July 2008 to July 2009). Three instruments that were used, included: Diagnostic and statistical manual of mental illness (DSM IV)[7] that is the most valid instrument for diagnosis of mental disorder; diagnostic criteria for psychosomatic research (DCPR)[8], that explores a variety of possible psychological conditions and emotional responses to medical illness; psychosocial index[9], that is a self-report instrument for assessing acute and chronic stresses, psychological distress, abnormal illness behavior, and psychological well-being. Patients were visited by researchers once a month during the follow-up visit in one year. Paired samples t-test and survival analysis were used. This study complies with the declaration of Helsinki. All participants declared their consent to participate before inclusion in the study.

Results about psychiatric diagnosis and psychosomatic diagnosis at the first assessment and after one year have been listed in Table 1. Three patients were taking chlordiazepoxide and two patients were taking lorazepam as prescribed at the follow-up visit. None of the patients had ever taken any psychoactive drugs or had ever undergone psychological treatments for anxiety and mood disorder. Results also indicated the mean scores of the psychosocial index that were: 6.0 (SD=4.8), for psychological distress, 1.8 (SD=2.8) for abnormal illness behavior, 1.9 (SD=2), for stress and 4.5 (SD=1.6), for psychological well-being for one month after ICD, and means scores: 7.0 (SD=4.9), for psychological distress, 0.7 (SD=0.8), for abnormal illness behavior, 1.4 (SD=1.5), for stress, 5.8 (SD=1.4), for psychological well-being at the fallow up assessment. After one year follow up, 8 patients had experienced myocardial infarction that three of them had died and 14 patients with angina pectoris had reported it at least once a month in the year. So, in 22 patients with coronary events, the following psychological symptoms were reported by 18 patients: 7 patients with obsessive compulsive disorder (OCD), 4 patients with OCD plus minor depression, 3 patients with minor depression, 2 patients with generalized anxiety, and 2 patients with social phobia. Thus, among psychological factors, OCD had maximum risk for coronary events (K-square=9.85, p=0.003), and other psychological factors that were related to coronary events (predictors of coronary events) were respectively OCD plus minor depression, minor depression, generalized anxiety and social phobia. It is important that among 36 patients, after one year follow up, 6 patients reported OCD with minor depression and 3 patients reported generalized anxiety with minor depression, so this finding indicates that there is positive relationship and comorbidity between anxiety disorders (such as OCD) and mood disorders (such as depression).

**Table 1:** First evaluation and follow-up study according to DSM–IV about psychiatric and psychosomatic diagnoses

**Psychiatric Diagnosis**	**Intake (N=36) % Diagnoses (N=15)**	**Followup (N=27) % Diagnoses (N=11)**	**Psychosomatic Diagnosis**	**Intake (N=36) % Diagnoses (N=22)**	**Followup (N=27) % Diagnoses (N=19)**
Obsessive compulsive disorder	26.66	18.18	Type A behavior	27.27	22.22
Somatoform disorder	20	9.09	Irritable mood	27.27	22.22
Generalized anxiety	13.33	9.09	Demoralization	18.18	16.16
Social phobia	13.33	18.18	Health anxiety	9.09	11.11
Minor depression	13.33	18.18	Illness denial	9.09	11.11
Major depression	6.66	9.09	Persistent somatization	4.54	5.55
Agoraphobia	6.66	9.09	Disease phobia	4.54	5.55
Bipolar disorder	0.0	0.0	Alexithymia	4.54	5.55
Panic disorder	0.0	9.09			

The present study offers new clinical approach to anxiety disorders after ICD and studying its effectiveness on cardiac events in Asian countries especially in Iran because of increased psychological stressors due to interpersonal, social and economical problems and so on in these countries. One year follow- up visit in this study revealed that most of the psychosomatic diagnoses were still present over this period of time in some patients according to DCPR, that lead us to conclude that the DCPR categories remain stable in individuals over time. Results also indicated that DSM based OCD is a risk factor for cardiac events, during one year after ICD. It is likely that OCD similar to other anxiety disorders is thought of as an adaptable and inevitable reaction to the effects of cardiac surgery [10][11] Also OCD is a disorder that accompanies with few disorders such as somatoform and physical disorders because patients with these disorders suffer from their thoughts about physical problems and these thoughts and distresses are considered as one of the symptoms of OCD [12].
